# Methodology and reporting quality of patient and public versions of guidelines in China: A systematic review

**DOI:** 10.1002/gin2.12024

**Published:** 2024-07-09

**Authors:** Hui Liu, Yuanyuan Yao, Nan Yang, Zijun Wang, Xufei Luo, Dongrui Peng, Huayu Zhang, Junxian Zhao, Hongfeng He, Xingrong Liu, Yaolong Chen, Janne Estill

**Affiliations:** ^1^ Research Unit of Evidence‐Based Evaluation and Guidelines (2021RU017), Chinese Academy of Medical Sciences, School of Basic Medical Sciences Lanzhou University Lanzhou Gansu China; ^2^ Key Laboratory of Evidence Based Medicine of Gansu Province Lanzhou Gansu China; ^3^ Institute of Health Data Science Lanzhou University Lanzhou Gansu China; ^4^ Evidence‐Based Medicine Center, School of Basic Medical Sciences Lanzhou University Lanzhou Gansu China; ^5^ School of Public Health Lanzhou University Lanzhou Gansu China; ^6^ Institute of Global Health University of Geneva Geneva Switzerland; ^7^ WHO Collaborating Centre for Guideline Implementation and Knowledge Translation Lanzhou Gansu China

**Keywords:** China, patient and public versions of guidelines, patient guidelines

## Abstract

**Introduction:**

The development of patient and public versions of guidelines (PVGs) in China is still in its early stages. The aim of this article is to systematically identify the PVGs published or released in China, analyse their development methods, and assess their reporting quality.

**Methods:**

We searched five major literature databases and conducted supplementary searches to identify all PVGs published or released by 8 January 2023 in China. After screening the literature according to the inclusion and exclusion criteria, we analysed the development methodology and evaluated the reporting quality of the included PVGs using the Reporting Items for Practice Guidelines in Healthcare‐Public or Patient Versions of Guidelines (RIGHT‐PVG). We reported this systematic review in compliance with the Preferred Reporting Items for Systematic Reviews and Meta‐Analyses (PRISMA) guidelines.

**Results:**

A total of 3795 records were first identified, and 17 PVGs were included. Nine PVGs reported their development methodology: seven used de novo development (similar to developing clinical practice guidelines [CPGs]), and two rewrote the recommendations of an existing CPG. The reporting quality differed substantially between the PVGs. The PVGs adhered to between 8 and 16 (47.1%–94.1%) of the 17 RIGHT‐PVG items, with a median of 9. All PVGs specified the topic addressed in the PVGs, introduced the target condition, described the purpose, scope and target users and had precise recommendations. In contrast, none of the PVGs listed questions for patients to ask their healthcare providers.

**Conclusions:**

Only few PVGs have so far been released in China. Most PVGs were developed de novo from the evidence, while some were instead rewritten from an existing CPG. In addition, we encourage PVGs developers to follow RIGHT‐PVG checklist when writing the guidelines in the future.

## Highlights

- Only few patient and public versions of guidelines (PVGs) have so far been published or released in China.
- Most PVGs in China were developed de novo from the evidence, while some were rewritten from an existing clinical practice guideline.
- The reporting quality varied substantially both between the PVGs and between the different items of Reporting Items for Practice Guidelines in Healthcare‐Public or Patient Versions of Guidelines (RIGHT‐PVG) checklist.

## 1 INTRODUCTION

Clinical practice guidelines (CPGs) provide effective guidance for healthcare practice, but they are usually written primarily for healthcare professionals.^1^, ^2^ Patients and the public are also important stakeholders in healthcare practice activities and thus should have access to in‐depth knowledge about the prevention, diagnosis, treatment and prognosis of diseases or clinical conditions. Relying on the principles of evidence‐based medicine, patient and public versions of guidelines (PVGs) have gradually gained attention and importance due to their professional approach, usefulness, credibility and function in shared decision‐making.^3^, ^4^, ^5^ As an example, in China, researchers have explored the methodology, principles and selection of topics for the development of PVGs^6^, ^7^, ^8^, ^9^ and also put forward some thoughts and suggestions based on the situation in China, such as discussing the development strategy and value of PVGs in the field of traditional Chinese medicine (TCM)^8^ and proposing reflections on the development of PVGs for the management of chronic diseases with TCM.^10^ The WHO Collaborating Centre for Guideline Implementation and Knowledge Translation at Lanzhou University, in collaboration with several Chinese institutions, organized the first methodological seminar on PVGs in Zhuhai, China, in May 2021, and initiatives were also proposed on promoting the development of high‐quality PVGs in China.^11^

However, a uniform definition of PVGs has not yet been established, and the development methodology has not been described in detail in most PVGs.^12^, ^13^, ^14^, ^15^, ^16^, ^17^, ^18^ Santesso et al. analysed 34 PVGs developed by 17 organizations and found that only 50% of the PVGs described, for example, what is the definition of a guideline or how the PVGs was developed.^16^ Guidelines International Network (GIN) published the *G‐I‐N Public Toolkit: Patient and Public Involvement in Guidelines* in 2015, which addresses the definition of PVGs, the workflow, principles and methodology of their development and the form of presentation.^19^ The German Agency for Quality in Medicine also published a manual for the development of PVGs in 2016, which provides an overview of the process and principles of PVG development.^20^ No academic institution in China has yet released a handbook for the development of PVGs, but some research groups have proposed methods of developing PVGs: for example, scholars from Tianjin University of Traditional Chinese Medicine and Beijing University of Traditional Chinese Medicine have explored the principles and processes of developing PVGs on the topics of nonpharmacological measures for secondary prevention of myocardial infarction and high risk of diabetic feet, respectively.^21^, ^22^

Clear and transparent reporting of guideline development methodology is critical to help users trust, use and disseminate PVGs. However, the status of the development methodology and reporting quality of PVGs from China has not been thoroughly assessed yet. Therefore, we aimed to conduct a systematic review to comprehensively analyse the development methodology and reporting quality of PVGs developed in China.

## 2 METHODS

We reported this systematic review in compliance with the Preferred Reporting Items for Systematic Reviews and Meta‐Analyses (PRISMA) guidelines.^23^

### 2.1 Information sources and search strategy

We searched Medline (via PubMed), Web of Science, China National Knowledge Infrastructure (CNKI), Wanfang Data Knowledge Service Platform (Wanfang) and China Biomedical Literature database (CBM) using search terms including “patient version*”, “patient guideline*”, “lay version*”, “public version*” and their derivatives. The details are presented in Supporting Information S1: Appendix 1. Two investigators (Hui Liu and Yuanyuan Yao) independently searched the five above‐mentioned databases on 8 January, 2023. We also consulted experts in the field and searched the official websites of major Chinese societies and associations, such as the Chinese Medical Association, the Chinese Physicians' Association and the Chinese Anti‐Cancer Association, as well as social media platforms, tracing references to relevant literature.

### 2.2 Inclusion and exclusion criteria

We included all PVGs developed in China without restrictions on the topic or the language of publication. We defined PVG as a guideline or consensus statement that fulfills all of the following criteria: (1) the document is developed by a medical professional institution, academic organization or expert group; (2) the target group is other than medical or healthcare professionals, such as patients and the general public and (3) the document is explicitly titled as nonprofessional version, containing terms such as “patient version”, “popular science version”, “patient guideline” or “patient and public guideline” in the title.

The following types of records were excluded: (1) PVGs protocols; (2) interpreted versions of actual PVGs; (3) PVG‐related news, reviews and methodological literature; (4) PVGs for which the full text was not available and (5) CPGs developed primarily for healthcare professionals whose target user groups also included patients or the public.

### 2.3 Study selection

All retrieved records were imported into EndNote 20 software. Two investigators (Hui Liu and Yuanyuan Yao) independently screened first the titles and abstracts and then the full texts of potentially relevant articles according to the inclusion and exclusion criteria. Disagreements were resolved through discussion and consultation with a third researcher (Nan Yang).

### 2.4 Data extraction

One investigator (Hui Liu) extracted the relevant information according to a predesigned extraction form, which was checked by another investigator (Yuanyuan Yao). We extracted the topic, year, platform of publication, leading organization/institution, development method (reported/not reported), quality of evidence, strength of recommendations, prospective registration (yes/no), participation of evidence‐based medicine (EBM) experts in the development (yes/no), participation of patients in the development (yes/no), inclusion of clearly formatted clinical questions (yes/no) and total number of recommendations. If the second investigator disagreed with the initial assessment, the disagreement was resolved through discussion. The development methods were further assessed qualitatively.

### 2.5 Quality assessment

The quality of guidelines generally includes two aspects: methodology and the comprehensiveness of reporting. We analysed the methodological quality only descriptively due to the lack of an appropriate assessment tool. For the assessment of reporting quality, the Reporting Items for Practice Guidelines in Healthcare (RIGHT) working group has developed an extension of the RIGHT for public or patient versions of guidelines (RIGHT‐PVG) in 2021.^24^ The checklist contains 17 items grouped into four domains. We slightly revised four items of RIGHT‐PVG in the assessment to be better applicable to PVGs using different development methods (Supporting Information S1: Appendix 2). Before the formal assessment, two investigators (Hui Liu and Yuanyuan Yao) conducted a pilot test to standardize the assessment criteria. The criteria were revised until the intraclass correlation coefficient was at least 0.9. During the formal assessment, the same two investigators conducted the assessment independently and cross‐checked the results. Any inconsistencies were resolved through discussion and consultation with a third investigator (Zijun Wang).

### 2.6 Data analysis

Microsoft Excel 2019 was used to collate the data and calculate the compliance rate (percentage of included PVGs that adequately reported the contents required in the item) for each RIGHT‐PVG item. We presented the results descriptively using frequencies and percentages.

## 3 RESULTS

### 3.1 Search results

**Figure 1:**
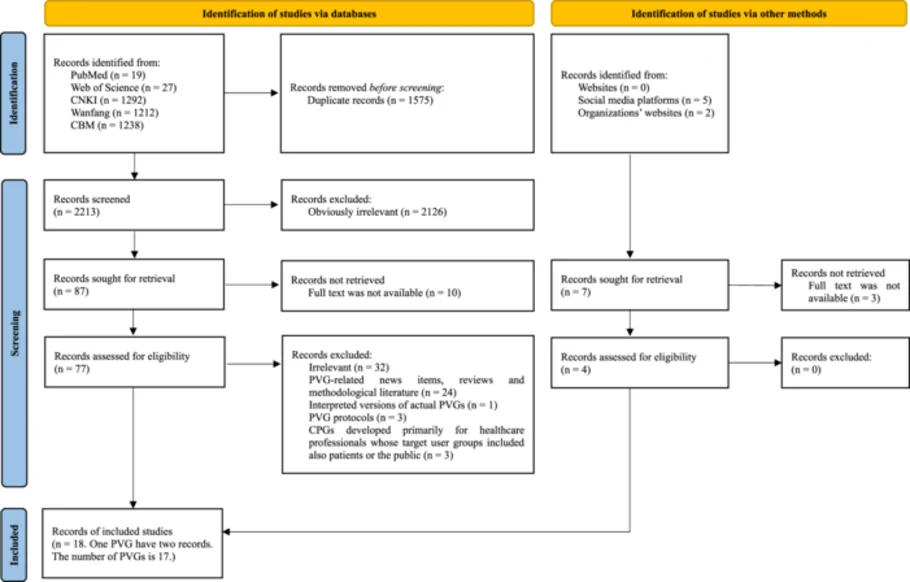
Literature screening flowchart.

A total of 3788 records were retrieved via databases, of which 1575 were excluded as duplicates. We screened the titles and abstracts of 2213 records, and, after excluding 2126 obviously irrelevant records, the full texts of the remaining 87 in strict accordance with the inclusion and exclusion criteria. After adding seven records identified from other sources, 17 PVGs were finally included.^25^, ^26^, ^27^, ^28^, ^29^, ^30^, ^31^, ^32^, ^33^, ^34^, ^35^, ^36^, ^37^, ^38^, ^39^, ^40^, ^41^, ^42^ All PVGs were written in simplified Chinese. The detailed screening process including the reasons for exclusion is shown in Figure 1.

### 3.2 Basic characteristics

Three (17.6%) of the 17 included PVGs were on TCM. Twelve (70.6%) PVGs were published in journals, three (17.6%) through social media, one (5.9%) on a website and one (5.9%) in a newspaper. Thirteen (76.5%) PVGs were developed by medical professional institutions and/or academic organizations and four by independent expert groups. Fourteen (82.4%) PVGs had clear clinical questions. However, only nine (52.9%) PVGs reported the methodology, seven (41.2%) PVGs assessed the quality of evidence and/or strength of recommendations, six (35.3%) PVGs were prospectively registered, eight (47.1%) involved EBM practitioners and eight (47.1%) involved patients, their families or members of the public in the development (Table 1).

**Table 1:** Basic characteristics of the patient and public versions of guidelines released in China (n=17)

Reference	Topic	Year	Publication platform	Leading developer organization/institution	Development method	Quality of evidence assessment
^[25, 26]^	Chronic gastritis (TCM)	2013	Newspaper	Expert panel	Not reported	Not reported
^[27]^	ankylosing spondylitis/spondyloarthritis	2020	Journal	Guangdong Provincial Clinical Research Center for Immunological Diseases, etc.	Reported (de novo development)	GRADE
^[28]^	Rheumatoid arthritis	2020	Journal	Guangdong Provincial Clinical Research Center for Immunological Diseases, etc.	Reported (de novo development)	GRADE
^[29]^	Osteoporosis	2020	Journal	Guangdong Provincial Clinical Research Center for Immunological Diseases, etc.	Reported (de novo development)	GRADE
^[30]^	Hyperuricemia/gout	2020	Journal	Guangdong Provincial Clinical Research Center for Immunological Diseases, etc.	Reported (de novo development)	GRADE
^[31]^	Myopia in children and adolescents (TCM)	2021	Journal	Ophthalmology Branch, Chinese Society of TCM	Not Reported	Not reported
^[32]^	Helicobacter pylori infection	2021	Journal	National Clinical Medical Research Center for Digestive Diseases (Shanghai), etc.	Reported (rewriting)	Not reported
^[33]^	Cough in children	2021	Journal	the Society of Pediatrics, Chinese Medicine Association	Reported (rewriting)	GRADE^a^
^[34]^	Fibromyalgia	2021	Journal	Chinese PLA Air Force Special Medical Center, etc.	Reported (de novo development)	GRADE
^[35]^	Ischemic stroke	2021	Journal	Experts panel	Reported (de novo development)	Not reported
^[36]^	Non‐alcoholic fatty liver disease (TCM)	2021	Journal	Experts panel	Not reported	Not reported
^[37]^	Primary liver cancer	2021	Website	China Anti‐cancer Association	Not reported	Not reported
^[38]^	Endometrial cancer	2021	Social media	China Anti‐cancer Association	Not reported	Not reported
^[39]^	Cervical cancer	2021	Social media	China Anti‐cancer Association	Not reported	Not reported
^[40]^	Ovarian cancer	2021	Social media	China Anti‐cancer Association	Not reported	Not reported
^[41]^	Tinea manus and pedis	2022	Journal	Expert panel	Not reported	Not reported
^[42]^	Foreign bodies in the digestive tract of children	2022	Journal	Chinese Society of Digestive Endoscopology	Reported (de novo development)	None^b^

Reference	Assessment of strength of recommendations	Prospective registration	EBM experts participated in development	Patients participated in development^c^	Inclusion of clearly formatted clinical questions	Total number of recommendations
^[25, 26]^	Not reported	No	No	None	Yes	‐
^[27]^	GRADE	Yes	Yes	Yes	Yes	15
^[28]^	GRADE	Yes	Yes	Yes	Yes	16
^[29]^	GRADE	Yes	Yes	Yes	Yes	14
^[30]^	GRADE	Yes	Yes	Yes	Yes	17
^[31]^	Not reported	No	No	No	No	‐
^[32]^	Not reported	No	No	No	Yes	16
^[33]^	GRADE^b^	No	Yes	Yes	Yes	29
^[34]^	GRADE	Yes	Yes	Yes	Yes	11
^[35]^	Not reported	No	Yes	Yes	Yes	54
^[36]^	Not reported	No	No	No	Yes	‐
^[37]^	Not reported	No	No	No	No	‐
^[38]^	Not reported	No	No	No	Yes	‐
^[39]^	Not reported	No	No	No	Yes	‐
^[40]^	Not reported	No	No	No	Yes	‐
^[41]^	Not reported	No	No	No	No	‐
^[42]^	Self‐designed	Yes	Yes	Yes	Yes	27

Abbreviations: EBM, evidence‐based medicine; GRADE, Grading of Recommendations Assessment, Development and Evaluation; TCM, traditional Chinese medicine; ‐, not applicable.

^a^ GRADE was used but presented in an adapted format.

^b^ Explanation for omitting the grading: "Considering that the users are patients and the public, it is not appropriate to make the content of the guidelines (including the methods for quality of the evidence and the strength of recommendations) overly complex and specialized, and after discussion among the experts on the methodology of the guidelines, it has been decided that the guidelines will not present quality of evidence, but only the strength of recommendations."

^c^ Patients include also the patient's family members and members of the public.

### 3.3 Development methodology

#### 3.3.1 Rewriting

Of the nine PVGs that reported the development methodology, two were developed by rewriting the recommendations of a single CPG. One of them was *Guidelines for Children with Cough in China (2021 Patients Version)*,^33^ which was developed from the source guideline *Clinical practice guidelines for the diagnosis and management of children with cough in China (2021 version)*.^43^ The CPG was published first, followed by the PVG with a 7‐month difference in submission and a 3‐month difference in publication dates. The number of recommendations increased from 28 in the source CPG to 29 in the PVG. Two recommendations in the PVG were new, one for the definition of the disease and one for the explanation of disease‐related terms. Both recommendations were also mentioned in the source CPG but not as recommendations. Moreover, two recommendations from the source CPG were merged together in the PVG. The assessment of the quality of evidence and strength of recommendations was also adapted from the source guideline that used the Grading of Recommendations Assessment, Development and Evaluation (GRADE) approach, but the symbols were transformed into smile and laugh to represent weak and strong recommendations, respectively, as well as numbers (1–4) to represent the quality of evidence. The readability of the rewritten recommendations was enhanced through focus group interviews. In addition, mind maps were added so that parents of affected children could grasp the core content of the PVGs more easily. The other PVG developed by rewriting was *Expert Consensus on the Prevention, Control and Management of Helicobacter pylori Infection in Chinese Residents' Families (Popular Science Edition, 2021)*,^32^ which was based on the source guideline *Expert Consensus on the Prevention, Control and Management of Helicobacter pylori Infection in Chinese Residents' Families (2021)*.^44^ The PVG was published 1 month after the source CPG and both had 16 recommendations. In addition to the substantial adjustments in the wording and presentation of the basis of the recommendations, the authors also added pictures (cartoons) to help users understand the content of the PVG.

#### 3.3.2 de novo development

Seven PVGs were developed de novo as independent guidelines, with a development process similar to that of CPGs. Six of the PVGs were developed in the following steps^27^, ^28^, ^29^, ^30^, ^34^, ^42^: establishing working groups, registration, writing the protocol, selecting and identifying clinical questions, retrieving evidence, evaluating and grading evidence and formulating recommendations and achieving consensus (Delphi survey). Five of these six PVGs used the GRADE system to grade the quality of evidence and strength of recommendations, while the remaining one presented only the strength of the recommendations in a self‐defined way (number of smiley faces) without assessing the quality of the evidence. One PVG developed de novo^35^ followed a slightly different process, which consisted of establishing the working group, collecting the health questions of concern to the patients, retrieving and evaluating relevant CPGs, extracting and integrating the recommendations from CPGs, convening a seminar for language translation and forming the first draft of the PVG and conducting an external review.

However, there were also some differences to the development process of CPGs. First, the process of forming clinical questions was different, and all PVGs conducted research on concerns of patients and/or the public. Second, guidelines and consensus statements were also included in the evidence retrieval and were treated as a type of evidence, unlike in CPGs that usually do not consider other guidelines and consensus statements as evidence. One PVG only included existing guidelines as evidence.^35^ Third, some guidelines took additional steps to ensure that the recommendations were understood by patients and the public. One PVG conducted investigations among representatives of patients and the public on the comprehensibility of the recommendations after they were formulated,^42^ and one convened a seminar for language translation.^35^

### 3.4 Assessment of reporting quality

**Figure 2:**
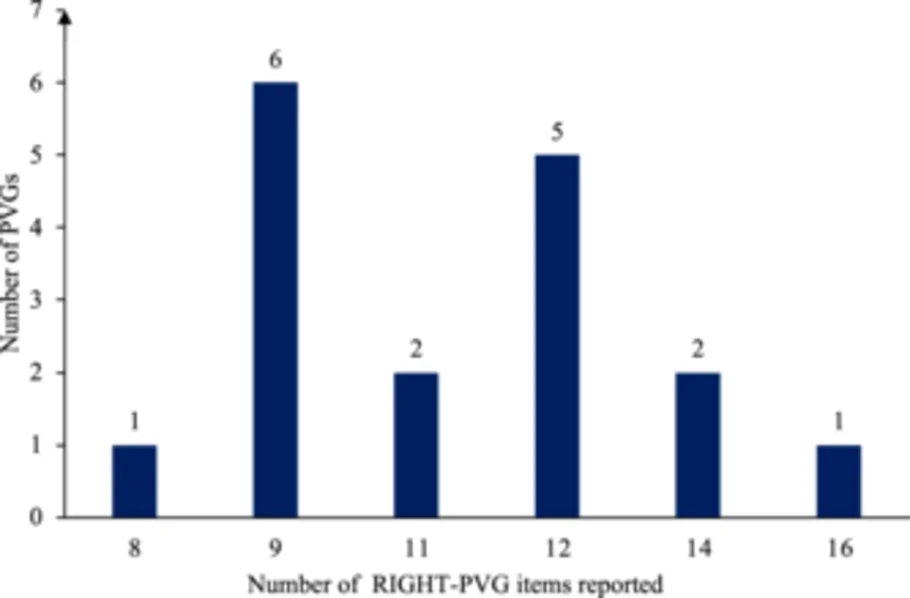
Distribution of the number of RIGHT‐PVG items adhered to by the PVGs. PVGs, patient and public versions of guidelines; RIGHT‐PVG, Reporting Items for Practice Guidelines in Healthcare‐Public or Patient Versions of Guidelines.

**Figure 3:**
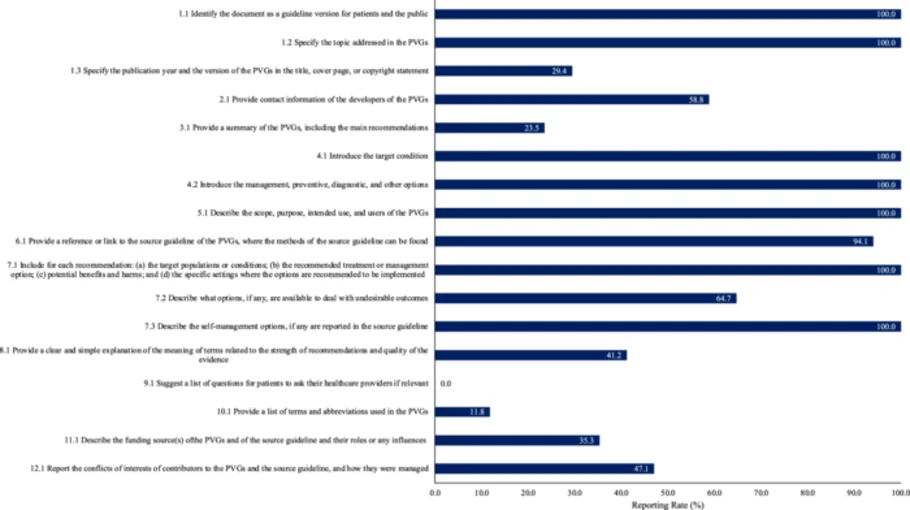
Compliance rate for each RIGHT‐PVG item. PVGs, patient and public versions of guidelines; RIGHT‐PVG, Reporting Items for Practice Guidelines in Healthcare‐Public or Patient Versions of Guidelines.

The PVGs adhered to between eight and 16 (47.1%–94.1%) out of the 17 items of RIGHT‐PVG items, with a median of nine. The compliance with different items varied also greatly: all PVGs adhered to item 1.1 (Identify the document as a guideline version for patients and the public), item 1.2 (Specify the topic addressed in the PVGs), item 4.1 (Introduce the target condition), item 4.2 (Introduce the management, preventive, diagnostic, and other options), item 5.1 (Describe the scope, purpose, intended use and users of the PVGs), item 7.1 (Include for each recommendation: a. the target populations or conditions; b. the recommended treatment or management option; c. potential benefits and harms; and d. the specific settings where the options are recommended to be implemented) and item 7.3 (Describe the self‐management options, if any are reported in the source guideline) and most (94.1% and 64.7%) also to items 6.1 (Provide a reference or link to the source guideline of the PVGs, where the methods of the source guideline can be found) and 7.2 (Describe what options, if any, are available to deal with undesirable outcomes), respectively. All other items were adhered to by less than 60% of the PVGs, with none of the PVGs being compliant with item 9.1 (Suggest a list of questions for patients to ask their healthcare providers if relevant; Figures 2 and 3).

## 4 DISCUSSION

### 4.1 Main findings

A total of 17 PVGs developed by Chinese institutions were included in our study. About half of the PVGs reported the development methodology, which we further categorized roughly into two types: rewriting, that is, transforming the recommendations of a CPG into a format that is easily understandable for patients and the public; and de novo development, that is, developing the PVGs independently with a process similar to that used to develop CPGs including a comprehensive search of the evidence, paying particular attention to the concerns of patients and the public. The reporting quality varied substantially both between the PVGs and between the different items of reporting.

### 4.2 Basic characteristics

Compared with the survey and analysis of PVGs in China conducted by Yan and other scholars,^45^ this study expanded the scope of the search by searching the official websites of major Chinese societies and associations, as well as social media platforms. We also expanded the search strategy and inclusion and exclusion criteria by including literature not explicitly named as “patient version” or “public version” but with other similar terms in the title, such as “popular science version” or “students' and parents' version”. Of the 17 PVGs published or released in China that we included, 80% were on Western medicine; the proportion is similar to the percentage of Western medicine guidelines and consensus statements among all guidelines published in China.^46^, ^47^ Easy and free access to patients and the public is a key facilitator in the successful dissemination of PVGs. Most PVGs published in countries other than China are released in PDF form through the official websites of the guideline developer organizations,^48^ whereas in China many PVGs are published primarily in academic journals, making it difficult to the patients to find and access them. We also found that some PVGs were released only as printed versions for purchase, and the full texts of some PVGs were even challenging to find. We thus recommend that the developers disseminate their PVGs through multiple channels and in multiple formats and not exclusively as journal articles so that the patients and the public can obtain the PVGs conveniently and free of charge. We also found that only about a third of the PVGs were registered before their development, which is much higher than the registration rate of CPGs.^46^, ^49^, ^50^ One reason may be that nearly half of PVGs included guideline methodologists, who are likely aware of the importance of registration, in their working groups.

### 4.3 Development methods

Both development methods, de novo development and rewriting, have their strengths and weaknesses.^51^ de novo development takes into full consideration the opinions and preferences of patients and the public at the stage of determining the clinical questions to ensure that the guideline addresses the true needs of patients and the public. This method, however, requires a substantial amount of time and resources. In contrast, development by rewriting takes a clearly shorter time which can accelerate the dissemination of knowledge about the disease. Translating published CPGs into PVGs can also efficiently support the dissemination and implementation of CPGs.^52^ However, considering that the clinical questions addressed by CPGs are primarily targeted at healthcare professionals, PVGs developed by rewriting may not be able to fully answer some common concerns faced by patients and the public.^33^, ^53^ The majority of PVGs developed elsewhere than in China have adopted the rewriting development method.^16^, ^17^, ^54^, ^55^ However, our study found that more than half of the Chinese PVGs that explicitly reported their development methods used de novo development, and rewriting was used by only few PVGs. There are several possible reasons for this difference. First, PVGs developers in different countries have differences in understanding and applying PVGs development methods. Second, some CPGs developers in China may not be aware of the importance of developing a corresponding PVGs, or there may be other reasons why it is not common in China to develop corresponding PVGs for CPGs. Third, PVGs may be developed by different institutions than the CPGs on the same topic, and PVGs developers may have chosen de novo development method to avoid intellectual property violations. Fourth, the developers of PVGs may believe that the scope of CPGs is too far from the topics of interest for the majority of patients and the public^33^ and therefore decide to develop PVGs de novo.

### 4.4 Reporting quality

The results of the assessment by RIGHT‐PVG showed that the reporting quality of PVGs in China still needs to be improved. For example, none of the PVGs provided a list of questions for patients to ask their doctors. The findings were similar to those on PVGs from settings other than China, with few providing lists of questions for patients to ask their doctors.^16^ RIGHT‐PVG was officially published in English in January 2021,^24^ and although its developers have introduced it in China through lectures, the Chinese translation of RIGHT‐PVG became available only in April 2022.^56^ The new edition (2022) of the guidance for development or revision of clinical diagnosis and treatment guidelines issued by the Chinese Medical Association explicitly mentions the recommendation for using RIGHT.^57^ The journals of the Chinese Medical Association and some other journals have included the RIGHT in the latest edition of their author guidelines, and editors also recommend RIGHT and different extensions when writing guidelines.^58^ Although the use of RIGHT and its extensions is to our knowledge not a mandatory requirement at the moment, we recommend to further publicize and promote RIGHT‐PVG to increase the awareness of it among PVGs developers and journal editors, which in turn can help to improve the reporting quality of PVGs. In addition, we also recommend that PVGs developers pay attention and focus on the simplicity and clarity of the language used in PVGs to make PVGs easier for patients and the public to read and use.

### 4.5 Advantages and limitations

Our study contains a comprehensive review of the PVGs published or released in China, including an evaluation of their development methodology and reporting quality. To ensure the accuracy of the results, the search, the literature screening and reporting quality assessment process were conducted by two investigators. The study has however still some limitations. First, the methodological quality of PVGs could not be assessed due to lack of an appropriate assessment tool at present, and some PVGs did not report the development methodology in detail. Second, we adapted some items of the RIGHT‐PVG tool to be better suitable to the different development methodologies, and our results may not be directly comparable with other studies that used RIGHT‐PVG. However, since the original version of RIGHT‐PVG only applies for PVGs developed by rewriting, our revision of the items can provide reference for future use of RIGHT‐PVG for assessing PVGs developed de novo.

### 4.6 Future research recommendations

To further promote the development of PVGs, we propose the following recommendations for future research. First, methodological manuals or guidance for the development of PVGs that are applicable to different countries or contexts should be developed. Second, methodological quality assessment tools for PVGs are needed. Third, RIGHT‐PVG should be translated into multiple languages and disseminated globally, and the checklist should be revised according to the local context and updated based on feedback from the users. Fourth, the current status of PVGs in different countries and regions needs to be comprehensively investigated.

## 5 CONCLUSIONS

Most PVGs from China were developed de novo, using methods similar to those used in the development of CPGs. The two main methods—de novo development and rewriting existing CPGs—have both their advantages and disadvantages, and PVG developers should be encouraged to carefully select the most appropriate method depending on their situation. A guidance for the development of PVGs in China is urgently needed. We also encourage PVGs developers to follow RIGHT‐PVG checklist when writing the guidelines to ensure that the PVGs can meet the diversified health needs of patients and the public.

## AUTHOR CONTRIBUTIONS

**Hui Liu**: Conceptualization; investigation; formal analysis; methodology; project administration; writing—original draft. **Yuanyuan Yao**: Investigation; formal analysis; visualization; writing—original draft; writing—review and editing; **Nan Yang**: Investigation; methodology; writing—review and editing. **Zijun Wang**: Investigation; Writing—review and editing. **Xufei Luo**: Writing—review and editing. **Dongrui Peng**: Writing—review and editing. **Huayu Zhang**: Writing—review and editing. **Junxian Zhao**: Writing—review and editing. **Hongfeng He**: Writing—review and editing; **Xingrong Liu**: Conceptualization; methodology; supervision; Writing—review and editing. **Yaolong Chen**: Conceptualization; methodology; funding acquisition; supervision, writing—review and editing. **Janne Estill**: Formal analysis; methodology; supervision; writing—review and editing.

## CONFLICT OF INTEREST STATEMENT

Hui Liu, Zijun Wang, Junxian Zhao, Xingrong Liu and Yaolong Chen participated in the development of *Management of foreign bodies in the digestive tract of children in China: a patient and public version guideline (2022)*. Hui Liu and Yaolong Chen participated in the development of *Guidelines for Children with Cough in China (2021 Patients Version)*. Yaolong Chen and Janne Estill participated in the development of RIGHT‐PVG (Reporting Items for Practice Guidelines in Healthcare‐Public or Patient Versions of Guidelines). And Yaolong Chen is the Co‐Founder and Co‐Chair of RIGHT. The remaining authors declare no conflict of interest.

## ETHICS STATEMENT

No ethics statement is required for this study, as it focuses on analyzing published literature.

## Supporting information

Supporting information.

## Data Availability

All relevant data are within the manuscript.
