# Supplementary material for: Methodology and reporting quality of patient and public versions of guidelines in China: A systematic review
Source: Clin Public Health Guidel. 2024 Jul 9;1(3):e12024. doi: 10.1002/gin2.12024 (PMC13563349; doi:10.1002/gin2.12024)
Supplement: Supplementary file 1 [file GIN2-1-e12024-s001.docx]

**Appendix 1**

Search Strategies for Chinese Patients and Public Versions of Guidelines

**PubMed**

#1. "guidelines as topic"[MeSH Terms]
#2. "Guideline"[Publication Type]
#3. "guideline*"[Title/Abstract]
#4. "guidance"[Title/Abstract]
#5. "guide"[Title/Abstract]
#6. "recommendation*"[Title/Abstract]
#7. "consensus"[Title/Abstract]
#8. OR/#1-#7
#9. "patient version*"[Title/Abstract]
#10. "patient guideline*"[Title/Abstract]
#11. "patients version*"[Title/Abstract]
#12. "patients guideline*"[Title/Abstract]
#13. "lay version*"[Title/Abstract]
#14. "public version*"[Title/Abstract]
#15. OR/#9-#14
#16. "public"[Title]
#17. "patient*"[Title]
#18. #16 AND #17
#19. #15 OR #18
#20. "China"[Affiliation]
#21. #8 AND #19 AND #20

**Web of science**

#1. TS=(guideline*)
#2. TS=(guidance)
#3. TS=(guide)
#4. TS=(recommendation*)
#5. TS=(consensus)
#6. OR/#1-#5
#7. TS=("patient version*")
#8. TS=("patient guideline*")
#9. TS=("patients version*")
#10. TS=("patients guideline*")
#11. TS=("lay version*")
#12. TS=("public version*")
#13. OR/#7-#12
#14. TI=(public)
#15. TI=(patient*)
#16. #14 AND #15
#17. #13 OR #16
#18. #6 AND #17
#19. CU=(China)
#20. #18 AND #19

**中国知网**

#1. 指南[篇名]
#2. 共识[篇名]
#3. #1 OR #2
#4. 患者[篇名]
#5. 公众[篇名]
#6. 家长[篇名]
#7. 科普[篇名]
#8. OR/#4-#7
#9. #3 AND #8

**万方**

#1. 指南[题名]
#2. 共识[题名]
#3. #1 OR #2
#4. 患者[题名]
#5. 公众[题名]
#6. 家长[题名]
#7. 科普[题名]
#8. OR/#4-#7
#9. #3 AND #8
#10. 选择"医药、卫生"分类

**中国生物医学文献数据库**

#1. "指南"[不加权:扩展]
#2. "指南"[中文标题:智能]
#3. "共识"[中文标题:智能]
#4. OR/#1-#3
#5. "患者"[中文标题:智能]
#6. "公众"[中文标题:智能]
#7. "家长"[中文标题:智能]
#8. "科普"[中文标题:智能]
#9. OR/#5-#8
#10. #4 AND #9

**Appendix 2**

RIGHT-PVG checklist

| Section/topic | **Items** |
| --- | --- |
| **Basic information** |  |
| 1 Title/cover/copyright | 1.1 Identify the document as a guideline version for patients and the public. |
|  | 1.2 Specify the topic (e.g., condition, technique, or medication) addressed in the PVGs. |
|  | 1.3 Specify the publication year and the version (if applicable) (e.g., first version, second version) of the PVGs in the title, cover page, or copyright statement. |
| 2 Contact information | 2.1 Provide contact information of the developers of the PVGs (e.g., affiliations, website, or address, phone number, or email address). |
| 3 Summary | 3.1 Provide a summary of the PVGs, including the main recommendations. |
| **Background** |  |
| 4 Introduction of the target topic | 4.1 Introduce the target condition, including (as relevant) the definition, risk factors, signs, subtypes, complications, staging (progress), and epidemiology. |
|  | 4.2 Introduce the management, preventive, diagnostic, and other options. |
| 5 Purpose, scope, and target users | 5.1 Describe the scope, purpose, intended use, and users of the PVGs. |
| 6 Link to the source guideline | 6.1 Provide a reference or link to the source guideline of the PVGs, where the methods of the source guideline (e.g., the evidence review and recommendation development process) can be found. ^a^ |
| **Recommendations** |  |
| 7 Recommendations | 7.1 Include for each recommendation: (a) the target populations or conditions; (b) the recommended treatment or management option (e.g., prevention plan, diagnostic strategy, or rehabilitation); (c) potential benefits and harms, especially those that are patients important; and (d) the specific settings where the options are recommended to be implemented |
|  | 7.2 Describe what options, if any, are available to deal with undesirable outcomes. |
|  | 7.3 Describe the self-management options, if any are reported in the source guideline. ^b^ |
| 8 The strength of the recommendations and certainty of the evidence | 8.1 Provide a clear and simple explanation of the meaning of terms related to the strength of recommendations and quality of the evidence (e.g., by using commonly understood symbols) |
| **Other information** |  |
| 9 Questions to ask | 9.1 Suggest a list of questions for patients to ask their healthcare providers if relevant. |
| 10 Terms and abbreviations | 10.1 Provide a list of terms and abbreviations used in the PVGs. |
| 11 Funding | 11.1 Describe the funding source(s) of the PVGs and of the source guideline and their roles or any influences, in the PVGs or guideline development processes, respectively. ^c^ |
| 12 Conflicts of interest | 12.1 Report the conflicts of interests of contributors to the PVGs and the source guideline in a format that the patients and the public can understand, and how they were managed. ^d^ |

PVGs: Patient and public versions of guidelines.

^a^: If the PVG is not based on a source guideline, but is developed *de novo*, the content of item 6.1 is adjusted to "Provide references to the evidence cited".

^b^: If the PVG is not based on a source guideline, but is developed *de novo*, the content of item 7.3 is adjusted to "Describe the self-management options".

^c^: If the PVG is not based on a source guideline, but is developed *de novo*, the content of item 11.1 is adjusted to "Describe the funding source(s) of the PVGs and the roles or any influence in the development process of the PVG".

^d^: If the PVG is not based on a source guideline, but is developed *de novo*, the content of item 12.1 is adjusted to "Report the conflicts of interests of contributors to the PVG, and how they were managed, in a format that the patients and the public can understand".
